# Hydroxylapatite‐collagen hybrid scaffold induces human adipose‐derived mesenchymal stem cells to osteogenic differentiation in vitro and bone regrowth in patients

**DOI:** 10.1002/sctm.19-0170

**Published:** 2019-12-13

**Authors:** Elisa Mazzoni, Antonio D'Agostino, Maria Rosa Iaquinta, Ilaria Bononi, Lorenzo Trevisiol, John Charles Rotondo, Simone Patergnani, Carlotta Giorgi, Michael J. Gunson, G. William Arnett, Pier Francesco Nocini, Mauro Tognon, Fernanda Martini

**Affiliations:** ^1^ Department of Morphology Surgery and Experimental Medicine, University of Ferrara Ferrara Italy; ^2^ Department of Surgery University of Verona Verona Italy; ^3^ Maria Cecilia Hospital, GVM Care & Research Cotignola Italy; ^4^ Private Practice, Arnett and Gunson Facial Reconstruction Santa Barbara California; ^5^ Department of Oral and Maxillofacial Surgery Loma Linda University Loma Linda California

**Keywords:** bone, hASC, hydroxylapatite/collagen, osteogenic differentiation

## Abstract

Tissue engineering‐based bone graft is an emerging viable treatment modality to repair and regenerate tissues damaged as a result of diseases or injuries. The structure and composition of scaffolds should modulate the classical osteogenic pathways in human stem cells. The osteoinductivity properties of the hydroxylapatite‐collagen hybrid scaffold named Coll/Pro Osteon 200 were investigated in an in vitro model of human adipose mesenchymal stem cells (hASCs), whereas the clinical evaluation was carried out in maxillofacial patients. Differentially expressed genes (DEGs) induced by the scaffold were analyzed using the Osteogenesis RT^2^ PCR Array. The osteoinductivity potential of the scaffold was also investigated by studying the alkaline phosphatase (ALP) activity, matrix mineralization, osteocalcin (OCN), and CLEC3B expression protein. Fifty patients who underwent zygomatic augmentation and bimaxillary osteotomy were evaluated clinically, radiologically, and histologically during a 3‐year follow‐up. Among DEGs, osteogenesis‐related genes, including BMP1/2, ALP, BGLAP, SP7, RUNX2, SPP1, and EGFR, which play important roles in osteogenesis, were found to be upregulated. The genes to cartilage condensation SOX9, BMPR1B, and osteoclast cells TNFSF11 were detected upregulated at every time point of the investigation. This scaffold has a high osteoinductivity revealed by the matrix mineralization, ALP activity, OCN, and CLEC3B expression proteins. Clinical evaluation evidences that the biomaterial promotes bone regrowth. Histological results of biopsy specimens from patients showed prominent ossification. Experimental data using the Coll/Pro Osteon 200 indicate that clinical evaluation of bone regrowth in patients, after scaffold implantation, was supported by DEGs implicated in skeletal development as shown in “in vitro” experiments with hASCs.

## Graphical abstract

Schematic representation of the study design conducted in human adipose stem cells (hASCs) and in maxillofacial patients. A, The biocompatibility and osteogenic markers proprieties of Coll/Pro Osteon 200 scaffold were measured on hASCs up to day 40. B, Patients who underwent zygomatic augmentation were evaluated clinically, radiologically, and histologically up to 36 months after surgery.

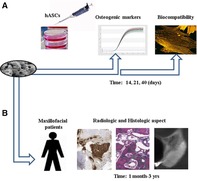

**Significance statement**

Bone regrowth can be achieved using different scaffolds. Biomaterials provide structural and biological cues to stem cells to stimulate the osteogenic differentiation. The new knowledge on the mechanisms of bone repair is of paramount importance to address significant steps needed in translational and precise medicine to cure patients. The hybrid scaffold Pro Osteon200/Avitene Collagen showed a significant osteogenic induction. The continuous supply of human adipose‐derived mesenchymal stem cells for bone regrowth/remodeling, chondrogenic, and osteoclast activities with their epigenetic modulations have been disclosed herein. The new data of this study indicate that the continuous expression of osteogenic, osteoclastic, and chondrogenic genes favors bone regrowth.

## 1 INTRODUCTION

Bone fractures/injuries can have a highly deleterious impact on patients' quality of life. Therefore, understanding the mechanisms of bone repair is important for both the patients' health and economy. Tissue engineering–based bone grafts are emerging as a viable treatment to repair and regenerate tissues damaged as a result of diseases. The structure and composition of scaffolds stimulate the classical osteogenic pathways through an active process which occurs in mesenchymal stem cells (MSCs). Investigators are facing a great challenge to design and develop suitable scaffold materials with biological activities for the tissue regeneration in translational and precise medicine. Tissue‐engineered approaches to regenerate bone in the craniomaxillofacial region utilize scaffolds to provide structural and biological cues to stem cells to stimulate osteogenic differentiation. The repair and regrowth of craniofacial tissues are particularly challenging, because they form a complex structure which is composed of hard and soft tissues involved in complex biological functions. Porous hydroxylapatite/collagen (HA/Coll) composite biomaterials are suitable for bone grafting, as well as for bone regeneration.^1^, ^2^

The biomaterial known as Pro Osteon 200 is a coral derived porous HA, which is available in granule and block forms. This scaffold has been shown to be highly biocompatible in cellular^3^, ^4^, ^5^ and animal models,^6^, ^7^, ^8^ and in some clinical applications.^9^ Scaffolds used for the bone regrowth can be assayed for specific features, such as osteoinductivity, osteoconductivity, and biocompatibility proprieties. Little is known about biological features and gene induction occurring after biomaterial adding.

In the past decade, significant studies have been carried out using human adipose‐derived stromal/stem cells (hASCs) in the bone tissue engineering regeneration/regrowth^10^ and tooth periodontal regeneration.^11^ hASCs are a type of adult MSCs capable of self‐renewal and differentiation toward osteogenic, chondrogenic, and adipogenic.^12^, ^13^, ^14^ Indeed, these cells own a multilineage differentiation capacity that is regulated through extracellular signals. The cellular events related to cell adhesion and cytoskeleton organization have been suggested as central regulators of differentiation fate decision. Little is known about the molecular mechanisms which allow hASCs to differentiate.^15^ Since the discovery of the hASCs induced‐osteogenesis, many studies substantially contributed the knowledge of hASC cell biology and their use as a cell source for bone regeneration.^16^ Ideal scaffolds, for hASC bone tissue engineering strategies, should be biocompatible and capable of supporting stem cells growth and differentiation, while providing predictable mechanical and degradation properties during the healing process.

In the present study, new investigations were carried out to evaluate the biocompatibility and osteoinductivity properties of the HA‐Coll hybrid scaffold in an “in vitro” model of hASC and in patients undergoing orthognathic surgical procedures associated with malar augmentation. The cellular biology, molecular genetic and epigenetic experiments were carried out by RT‐qPCR Array and protein expression, such as osteocalcin (OCN) and alkaline phosphatase (ALP) by immunostaining and ELISA approaches, respectively. The osteogenesis potential of cells was also investigated to verify the matrix mineralization by Alizarin‐Red S staining. The biocompatibility of the material was evaluated by cellular viability, morphology, and cytoskeleton architecture, in hASCs grown on the hybrid scaffold.

In a previous study^17^ gene expression analyses by RT‐PCR technology were carried out at early times post‐cell seeding on biomaterial. To this end, mRNAs extracted from cells, hASCs, were comparatively analyzed by RT‐PCR at days 3 and 9. hASCs were grown on the scaffold, and plastic vessel (TCPS), used as control. PCR technology demonstrated that biomaterial induces in hASCs upregulation of specific genes, such as SPP1 and CLEC3B, which are involved in bone mineralization and ossification processes, at days 3 and 9 post‐cell seeding. In addition, in our experimental conditions, ALP, ON, CHAD, SP7, and Sox9 genes had a higher expression compared with hASCs grown on TCPS, at day 3 post‐seeding.

In the present study, in order to mimic the long period needed “in vivo” by the bone to regrowth/repair/healing, the experiments were carried out up to day 40.

Patients operated for maxillomandibular malocclusion and/or asymmetry, or for aesthetic reasons, who underwent malar augmentation with porous HA/Coll prostheses (hybrid scaffold), were evaluated for the new bone formation during a 3‐year period of follow‐up using radiological and histological analyses.

The strength of this article is the combination of in vitro and in vivo evaluations of HA‐Coll hybrid scaffold on osteoinductivity and bone regrowth proprieties. In hASCs grown on biomaterial, RT^2^ Profiler PCR array “Human Osteogenesis” approach allowed us to analyze the epigenetic profiles of 84 genes belonging to the osteogenetic pathway, at two experimental time points, days 21 and 40.

## 2 MATERIALS AND METHODS

### 2.1 Experimental design

Biocompatibility, osteoinduction, and osteoconductivity proprieties of the innovative scaffold composed by Avitene Microfibrillar Collagen Hemostat and Granular Pro Osteon 200 coralline HA (Coll/Pro Osteon 200) were analyzed in hASC grown on the scaffold up to day 40. The clinical radiological and histological analyses were performed on orthognathic surgical patients during a 3‐year follow‐up (see the Graphical Abstract).

### 2.2 Cell culture

hASCs were purchased from Lonza Milan, Italy (PT‐5006) as cryopreserved frozen cells at the first passage. These cells are positive for surface markers CD13, CD29, CD44, CD73, CD90, and CD105, CD166, whereas they are negative for other markers, such as CD14, CD31, and CD45. Cells were expanded in Dulbecco's Modified Eagle Medium F‐12 (DMEM/F12; Lonza, Milan, Italy), as previously described.^17^, ^18^, ^19^ Primary hASC cultures were grown (i) in the presence of the osteogenic condition (OC) only^17^, ^20^; (ii) on the biomaterial Coll/ Pro Osteon 200^17^; (iii) in the tissue culture polystyrene (TCPS) vessels, the control.^17^ Experimental time points were at days 14, 21, and 40 as indicated for the different assays later in the article.

### 2.3 Biomaterial

Porous HA‐derived scaffold used herein is composed by Granular Pro Osteon200 (Interpore Cross Irvine, California) and Avitene Microfibrillar Collagen Hemostat (Bard Warwick, Rhode Island) (Coll).^9^, ^17^ This scaffold is named Coll/Pro Osteon 200. Granular Pro Osteon 200 is a coralline HA, which is very similar in its make up to human bone mineral composition and form. The manufacturing scaffold using in vitro and in vivo evaluations was descried before in detail.^9^, ^17^

### 2.4 Osteogenesis RT^2^ profiler PCR array

To identify genes of the osteogenic pathway activated by the scaffold, an osteogenesis PCR array was performed, in triplicate, in hASCs grown on the biomaterial. Specifically, total RNA was isolated through RNeasy Plus Micro Kit (Qiagen, Milan, Italy) according to the manufacturer's instructions from cells grown on (i) Coll/Pro Osteon 200 scaffold and (ii) TCPS (control group). RNA was quantified by using a Nanodrop spectrophotometer (ND‐1000; NanoDrop Technologies, Wilmington, Delaware).^17^, ^21^ The Human Osteogenesis RT^2^ Profiler PCR Array (Qiagen) was used as described.^17^, ^22^ Specific primers sets used in real‐time PCRs were used to analyze the expression of 84 genes involved in different pathways, such as osteogenic differentiation, cartilage condensation, ossification, bone metabolism, bone mineralization, binding to Ca^2+^ and homeostasis, extracellular matrix (ECM) protease inhibitors, adhesion molecules, cell‐to‐cell adhesion, adhesion molecules of the ECM, and growth factors. For data analysis, the fold change (FC) of each gene expression was calculated by using the 2^−ΔΔCt^ method, whereas housekeeping genes, used as controls, were used to normalize results and Log_2_ FC; <1 or >1 was considered significant.^17^, ^23^

### 2.5 Cell differentiation and matrix mineralization

Osteogenic proteins including Tetranectin/CLEC3B expression,^17^ ALP activity,^17^ and OCN expression levels were analyzed in hASCs grown on scaffold. ALP activity was determined, at day 40, by a colorimetric Naphthol AS‐BI phosphate‐based reaction using the Alkaline Phosphatase Detection Kit (Merck Millipore Corporation, Milan, Italy), following the manufacturer's indications, as described.^17^ Alizarin Red (Sigma, Milan, Italy, A5533) was used to analyze the matrix mineralization, as described.^17^, ^18^ The mineralized substrates were quantified by using a 20% methanol and 10% acetic acid in a water solution (Sigma‐Aldrich, Milan, Italy).^24^. To quantify the matrix mineralization, the solution was transferred into cuvettes, whereas the quantity of Alizarin red dissolved was read spectrophotometrically (Thermo Electron Corp., model Multiskan EX, Vantaa, Finland) at a wavelength (λ) of 450 nm. At days 14, 21, and 40, the expression of the OCN, as a protein, was investigated in hASCs grown in OC, on the biomaterial and TCPS. The protein was extracted with Cell Extraction Buffer (Catalog number FNN0011 Thermo Fischer Scientific, Milan, Italy) added with 1 mM phenylmethylsulfonylfluoride and a protease inhibitor cocktail. The concentration of total protein was determinate by bicinchoninic acid assay according to manufacturer's instructions.^19^ The OCN protein was quantified by the Human Osteocalcin Instant ELISA (Thermo Fisher Scientific, Milan, Italy) according to the manufacturer's instructions. Immunostaining was performed for the CLEC3B using the CLEC3B‐specific mouse monoclonal primary antibody (HYB 130‐11‐02, Thermo Fisher Scientific, Milan, Italy) and an anti‐mouse IgG (whole molecule) secondary antibody‐tetramethyl‐rhodamine‐isothiocyanate (TRITC), diluted in saline with 0.1% bovine serum albumin.^17^

### 2.6 Cell viability and cytoskeleton architecture

Viability of hASCs grown on the biomaterial was evaluated by fluorescent microscopy analysis using hASCs transfected with an adenovirus (Ad) vector expressing the green fluorescence protein (eGFP),^17^, ^25^ hASCs‐eGFP. Cytoskeletal actin filaments of hASCs‐eGFP were stained with TRITC‐conjugated Phalloidin (Sigma, Milan, Italy) as previous descried at days 14, 21, and 40.^17^, ^18^, ^19^

### 2.7 Scanning electron microscopy analysis

Biomaterial with and without cells was analyzed by scanning electron microscopy (SEM; Cambridge UK, model Stereoscan S‐360).^17^ Samples were washed with saline; then fixed for 1 hour by 2.5% glutaraldehyde and additional 4 hours with a 1% osmium solution in phosphate buffer. Then, the biomaterial was coated with colloidal gold and SEM analyzed.^17^, ^18^

### 2.8 Fluorescent and confocal microscope analyses

Fluorescent images were taken using a TE2000E fluorescence microscope. Digital images were captured using ACT‐1 and ACT‐2 software for DXM1200F digital cameras (Nikon S.p.A., Florence, Italy). Nuclei were stained with 0.5 mg/mL 4,6′‐diamino‐2‐phenylindole (DAPI).^26^, ^27^ Digital images of cellular morphology were acquired with confocal microscope (LSM510; Carl Zeiss, Jena, Germany) using a 10 × 1.4 NA Plan‐Apochromat oil‐immersion objective and equipped with ZEN microscope imaging software (Zeiss Instruments). The background of acquired images were corrected, whereas signals were analyzed using the open source Fiji software (http://fiji.sc/Fiji).

### 2.9 Bone regrowth evaluated in maxillofacial patients

The treatment plan, which aimed to achieve functional and esthetic improvement, was to enhance the malar area of patients with inadequate cheekbone projection or facial asymmetry. All orthognathic surgical procedures were carried out as previously described^9^ in the Arnett‐Gunson Center for Corrective Jaw Surgery (Santa Barbara, California) or at the Maxillofacial Surgery Unit of the University of Verona Medical Center (Verona, Italy).^9^Over a 3‐year period (2016‐2018), 50 patients (25 women and 25 men; mean age, 28 years; range, 18‐45 years, total 100 prosthesis) underwent malar augmentation procedures using the hybrid scaffold Collagen/Pro Osteon to optimize facial esthetics. The follow‐up time was 3 years. This study was approved by the Institutional Review Board of the Verona Hospital (Verona, Italy). All patients signed an informed written consent agreement. The present study followed the Declaration of Helsinki on medical protocol.

#### 2.9.1 *Radiologic imaging*

CT scans taken 1 month (T1) and 24 months (T2) after surgery were available for all patients. Signs of resorption of native bone and variations in the structure and radiopacity of the implanted prosthetic material were evaluated by an observer, in a blind manner (the radiologist who analyzed the cone‐beam computed tomography—CBCT). Twenty of 50 patients also agreed to undergo a CBCT (NewTom 3G device; QR srl, Verona, Italy) at least 36 months after surgery (T3).

#### 2.9.2 *Histology*

Biopsy samples were obtained from implants in three patients who required plates removal at maxillae level 2 years after surgery. The material collected was treated as described.^9^ Morphologic and immunohistochemical analyses of bone samples were performed to evaluate remodeling and osteogenetic process. Four‐micrometer‐thick sections were stained with hematoxylin and eosin, and immunohistochemistry was performed on subsequent sections using two monoclonal antibodies specific for cathepsin K (clone CK4, Novocastra, Newcastle, UK; clone 3F9, Abcam, Cambridge, UK) to stain osteoclasts and for CD56 (clone 123C3.D5, 1:100; Thermo Scientific, Grand Island, New York) to stain osteoblasts, as previously described.^9^

### 2.10 Statistical analysis

Statistical analyses of experiments, performed in triplicate, were carried out by using Prism 6 (GraphPad Software, La Jolla, California),^23^, ^28^, ^29^ one‐way analysis of variance with Dunnett's posttest analysis and Student's *t* test. A value of *P*‐value <.05 was considered significant.

## 3 RESULTS

### 3.1 In vitro and in vivo evaluations

Biocompatibility, osteoinductivity, and osteoconductivity properties of the innovative scaffold composed by Avitene Microfibrillar Collagen Hemostat and Granular Pro Osteon 200 coralline HA (Coll/Pro Osteon 200) were analyzed in hASC grown on the scaffold up to day 40. The clinical radiological and histological analyses were performed on orthognathic surgical patients during a 3‐year follow‐up (see Graphical Abstract for more details).

### 3.2 Scaffold modulates the DEGs implicated in skeletal development

**Figure 1:**
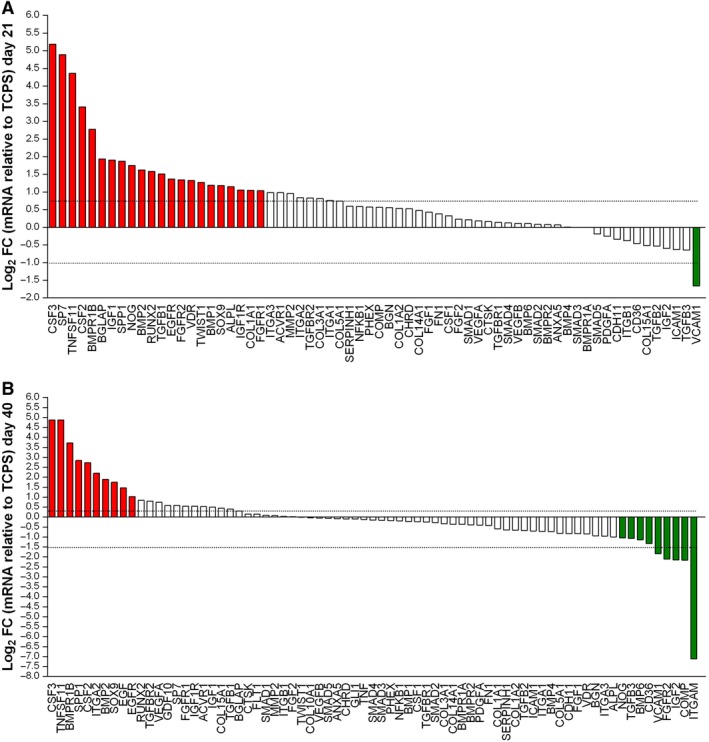
PCR array analyses of osteogenic genes. The gene expression was evaluated in human adipose mesenchymal stem cells grown on Coll/Pro Osteon200 compared with tissue culture polystyrene. A, Induced genes at day 21. In hASC cultures, mRNAs of 22 genes of the osteogenic pathway, that is, ALPL BGLAP BMP1/2, BMPR1B, COL1A1,CSF2/3, EGFR, FGFR1, FGFR2, IGF1, IGF1R, NOG,RUNX2, SOX9, SP7, SPP1, TGFB1, TNFSF1, TWIST1, and VDR were upregulated; one gene, such as VCAM1 was downregulated. B, Induced genes at day 40. In hASC culture, mRNAs of 10 genes of the osteogenic pathway, that is, BMP2, BMPR1B, CSF2, CSF3, EGF, EGFR, ITGA2, SOX9, SPP1, and TNFSF11 were upregulated, whereas nine genes were downregulated (BMP6, CD36, COMP FGFR2, IGF2, ITGAM, NOG TGFB3, and VCAM1)

**Figure 2:**
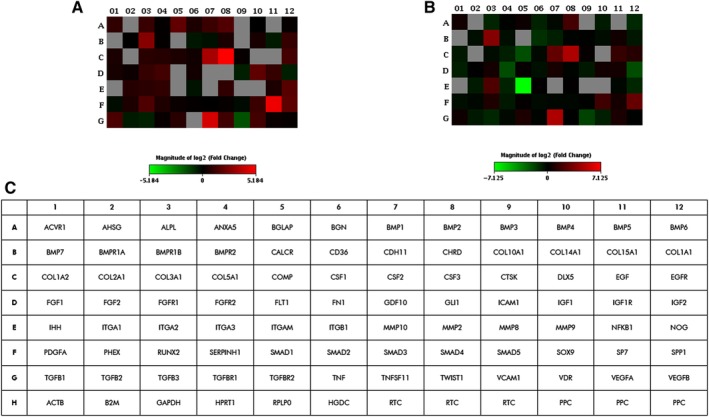
Graphical representation through heat map of the mRNA expression in human adipose mesenchymal stem cells (hASCs) grown on Coll/Pro Osteon 200. The fold‐change values of upregulated (red) and downregulated (green) genes in hASCs grown on Coll/Pro Osteon 200 compared with the control are reported at day 21 (A) and day 40 (B), respectively. C, Plate scheme of RT^2^ osteogenic array

In a previous study, we investigated in a short period of time few specific osteogenic genes, such as ALP, Osteonectin, Transcription factor Osterix, SP7 and CLEC3B, which were reported upregulated in hASCs grown on the scaffold, at day 9 (24). Herein, RT^2^ Profiler PCR array was used to analyze the expression of osteogenic genes. The gene expression was evaluated in hASCs grown on Coll/Pro Osteon200 compared with TCPS, at days 21 and 40. A total of 31 differentially expressed genes (DEGs) including 22 upregulated genes (red), 7 downregulated genes (green), and 2 genes which were upregulated (day 21) and then downregulated (day 40), were identified in hASCs grown on the biomaterial (Figure 1A, B; Tables S1 and S2). Among DEGs, osteogenesis‐related genes, including the Bone Morphogenetic Protein 1/2 (BMP1/2), ALP, Bone Gamma‐Carboxyglutamate Protein (BGLAP), transcription factor Sp7 (SP7), Runt‐related transcription factor 2 (RUNX2), Secreted Phosphoprotein 1 (SPP1), Collagen type I alpha 1 (COL1A1), epidermal growth factor receptor (EGFR), which play important roles in osteogenesis, were found to be upregulated at day 21. Moreover, the transcription factor condensation SRY (sex‐related Y)‐type high mobility group box SOX‐9 (Sox9), and BMPR1B which plays a central role in chondrocyte differentiation, were also found to be upregulated, on hASCs grown on the scaffold, at days 21 and 40. Real‐Time qRT‐PCR revealed increased expression of the receptor activator of nuclear factor kappa‐Β ligand (RANKL), also known as tumor necrosis factor ligand superfamily member 11 (TNFSF11) at days 21 and 40. The growth factors, such as the colony‐stimulating factor 2/3 (granulocyte‐macrophage) (CSF2/3), and epidermal growth factor (EGF), were also found to be upregulated at days 21 and 40. The heat map, shown in Figure 2, provides a visualization of FCs of expression values among genes, in the array in the context of the array layout. hASCs grown on the biomaterial DEGs downregulated were n = 1 at day 21 and n = 9 at day 40. Genes encoding for cell‐ECM, adhesion molecules such as CD36 molecule thrombospondin receptor (CD36), Cartilage oligomeric matrix protein (COMP), and Integrin, alpha M complement component 3 receptor 3 subunit (ITGAM) were downregulated at day 40. The list of upregulated and downregulated genes at days 21 and 40 is reported (Table S1, Table S2), respectively.

### 3.3 Scaffold induces the matrix mineralization and osteogenic expression proteins in hASCs

**Figure 3:**
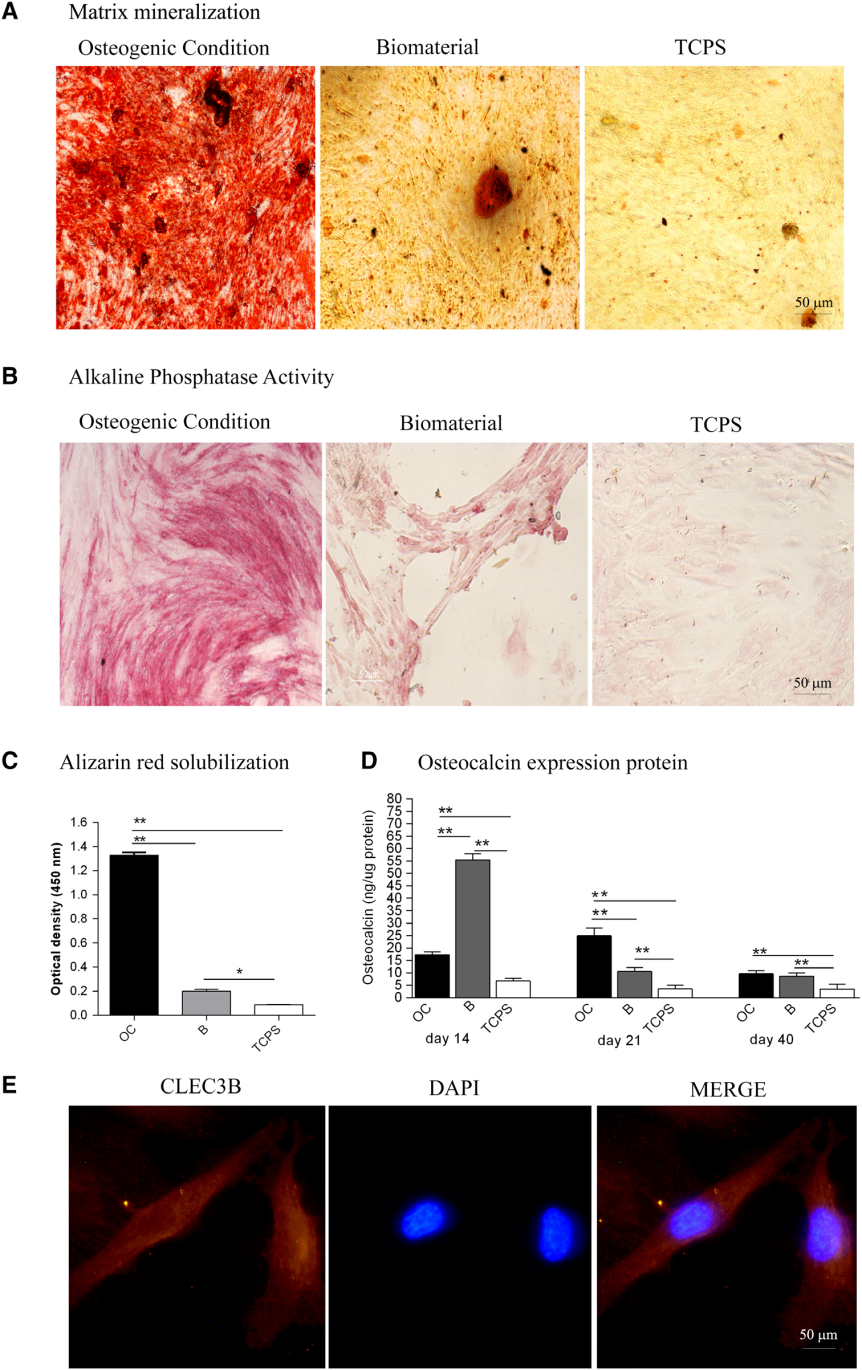
Osteogenic markers in human adipose mesenchymal stem cells (hASCs) cultured on the biomaterial. A, Alizarin red staining at day 40 is shown in the panel, in experimental conditions tested. Scale bar: 50 μm, Magnification ×4. B, Alkaline phosphatase (ALP) activity at day 40. Scale bar: 50 μm, Magnification ×4. C, The matrix mineralization was evaluated by Alizarin red staining, whereas its quantification was carried out spectrophotometrically. Matrix mineralization data were reported as optical density. Data are shown in the graph. D, The temporal pattern of osteocalcin (OCN) protein levels detected at different time points, that is, at days 14, 21, and 40, was quantified by ELISA. Osteocalcin protein was reported as nanograms of OCN/1 μg of total protein. E, Detection of C‐type lectin domain family 3, member B (CLEC3B) protein by immunostaining in hASCs, at day 40. Symbols indicate statistical significance (*P < .05; **P < .0001). Scale bar: 50 μm, Magnification ×40

Recently, we reported a significant increase of matrix mineralization and ALP activity in hASCs grown on the biomaterial, at day 21.^17^ In the present investigation, the osteoinductive activity of the biomaterial is highlighted by the matrix mineralization detected in hASCs grown on the scaffold at day 40. Indeed at day 40, the biomaterial favored the matrix mineralization better than the plastic vessel (TCPS), the control (Figure 3A,C, *P < .05). Moreover, calcium deposits in hASCs grown on OC were higher compared with cells grown on the biomaterial or TCPS (**P <. 0001; Figure 3A,C). Cells grown on the biomaterial and in OC showed a significant increase of the ALP activity compared with TCPS, at day 40 (Figure 3B).

ELISA data show a statistically significant increase of the OCN protein expression in cells grown on biomaterial, at the three time points, that is, at days 14, 21, and 40, compared with the control. This result is in agreement with previous data obtained at day 9.^17^ The expression of OCN in hASCs grown on the biomaterial was higher than in OC/TCPS, at day 14 (*P < .05). The hybrid scaffold influences the osteogenic pathway at days 21 and 40 compared with TCPS (*P < .05) (Figure 3D). Cells grown in OC showed higher expression levels of OCN compared with TCPS at days 14 and 21 (*P < .05) (Figure 3D).

CLEC3B/tetranectin protein was detected by immunohistochemistry in hASCs cultured on scaffold and in OC, at day 40 (Figure 3E), whereas it was absent in hASCs grown on TCPS (data not shown). CLEC3B protein, which binds Ca^2+^, was investigated because of its potential involvement in the bone mineral metabolism. In hASCs CD105‐enriched cell populations, an increased CLEC3B expression was detected in response to osteoinduction.^30^ In an earlier investigation, the CLEC3B mRNA expression levels tested upregulated at days 3 and 9.^17^

### 3.4 Scaffold is biocompatible in hASCs

**Figure 4:**
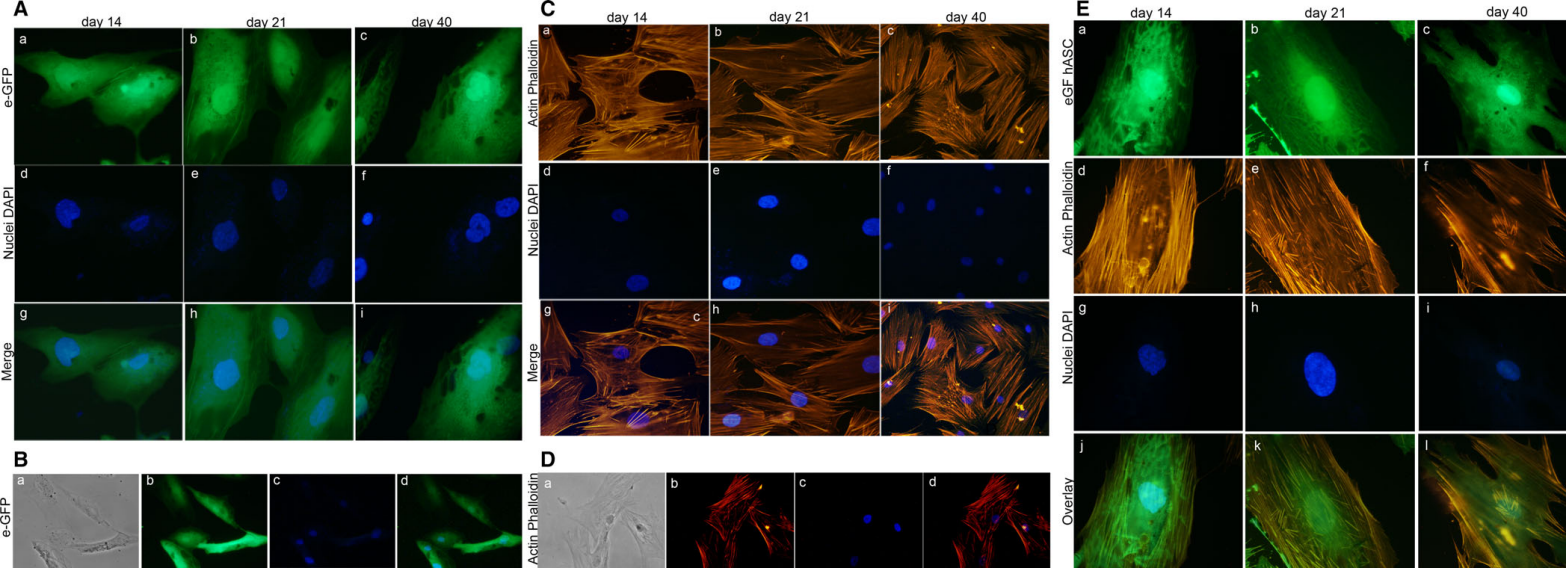
Stem cell viability and cytoskeleton architecture assays. A, Human adipose mesenchymal stem cell (hASC)‐eGFP grown on the biomaterial at days 14, 21, and 40 are shown at magnification ×40. B, hASC‐eGFP grown on the biomaterial at days 14, 21, and 40 are shown at magnification ×20. C, Cytoskeleton analysis by Phalloidin tetramethyl‐rhodamine‐isothiocyanate (TRITC) staining of hASCs grown on the biomaterial. Actin filaments do not show alteration in the structural organization, confirming the compatibility of the assayed biomaterial, at days 14, 21, and 40 (magnification ×40). D, Cytoskeleton analysis carried out by the confocal microscopy at day 40, magnification ×40. E, Cytoskeleton analysis by phalloidin TRITC staining of hASCs‐eGFP grown on the biomaterial, at days 14, 21, and 40 (magnification ×40). Cellular nuclei were stained with 0.5 mg/mL DAPI

hASCs grown on the biomaterial were investigated for their viability, proliferation, and cytoskeleton organization at days 14, 21, and 40. hASC‐eGFP grown on biomaterial showed a normal cell morphology (Figure 4A, B, E).^17^ The biomaterial demonstrated its biocompatibility up to day 40 in terms of cell adhesion and proliferation. hASC‐eGFP cell morphology was indistinguishable from parental hASCs (Figure 4A, B, E). The cytoskeleton architecture appeared to be well organized, whereas its integrity remains uninfluenced by the scaffold, up to day 40 (Figure 4C, E). Actin fibers seem to connect the cellular membranes and the cytoskeleton to the scaffold surface with no visible loss or structural displacement. Similar physiologic cytoskeleton architecture was observed by confocal microscopy at day 40 (Figure 4D).

### 3.5 SEM analysis

**Figure 5:**
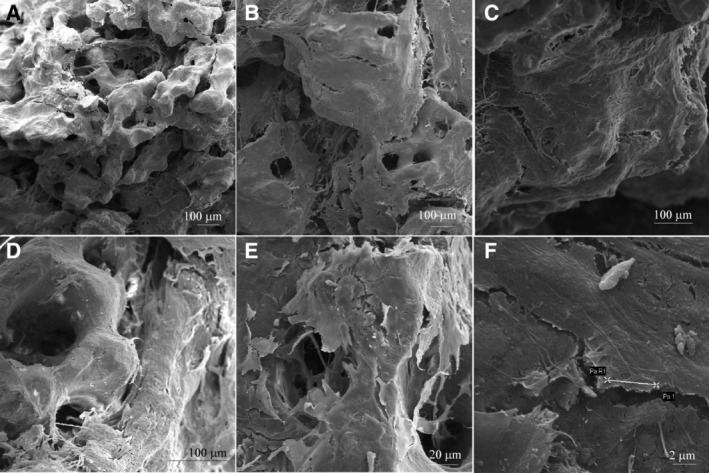
Scanning electron microscopy analysis of the Coll/Pro Osteon 200. A, Bovine collagen fibrils from Avitene Microfibrillar Collagen Hemostat were mixed with Granular Pro Osteon 200 to generate the scaffold Coll/Pro Osteon 200. The biomaterial shows the porous structure with several pores in the range 190 to 230 μm, Scale bar: 100 μm, ×86. B‐E, Human adipose derived stem cells (hASC) grown on HA‐derived scaffold for 40 days. The structure of scaffold was observed at different magnification, Scale bar: 100 μm, ×133 (B), Scale bar: 100μm, ×214 (C), Scale bar: 100 μm, ×344 (D), Scale bar: 20 μm, ×647 (E), respectively. F, Cells, homogeneously spread on the substrate, exhibited cytoplasmic bridges, whereas their morphology did not show any sort of alteration. Scale bar: 2 μm, magnification ×6.55k

SEM analysis was performed to investigate the microstructure and morphology of the scaffold (Coll/Pro Osteon 200) without hASCs (Figure 5A) and with cells grown on it, up to day 40 (Figure 5B‐F). The granular HA mixed with collagen fibers creates a highly fibrous structure. The biomaterial showed a different like‐bone structure, in the presence of cells, at day 40. hASCs grown on the scaffold showed a normal cell morphology exhibiting pseudopodium‐like structures in tight contact with the ECM (Figure 5F).

### 3.6 Bone regrowth in maxillofacial patients

**Figure 6:**
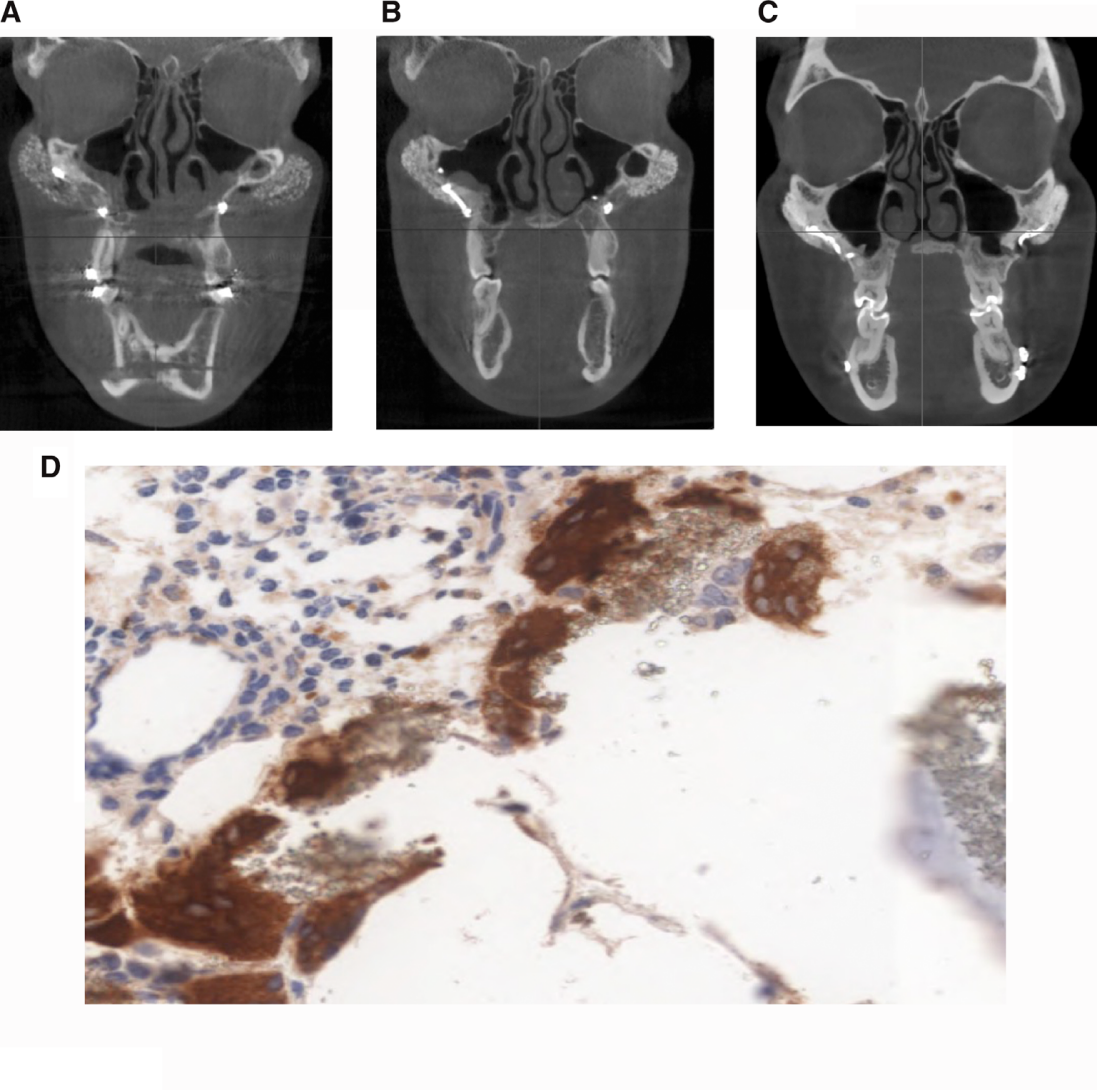
Scaffold characterization in patients: radiologic and histologic analyses. A‐C, Cone‐beam tomogram, coronal slice, at T1 (1 month), T2 (24 months), and T3 (36 months) after surgery. A, The prosthesis maintained its granular structure, whereas the granules did not migrate to the surrounding soft tissues. The structure of the prosthesis is radiotransparent compared with the compact portion of the zygomatic bone (T1). B, Cone‐beam tomogram, coronal slice, at 24 months after surgery. The prosthesis seems to adhere strongly to the underlying zygomatic bone in patients. The granular structure is still distinguishable, although less evident, whereas the partial radiotransparency evolved to a radiopacity similar to that seen in the compact part of the native bone, making it impossible to distinguish the interface between the prosthesis and bone. C, Cone‐beam tomogram, coronal slice, at 36 months after surgery. Progressive loss of definition of the granular architecture, with an almost complete radiopacity and apparent corticalization of the bone in contact with the prosthesis. The interface between the prosthesis and bone at T3 appears indistinguishable. D, Biopsies harvested 24 months after implant placement. Bone maturation gradient can be observed proceeding from the periosteal layer toward the native bone (hematoxylin and eosin stain: magnification ×10). Osteoclasts surrounding hydroxylapatite residual granules (immunohistochemistry with cathepsin K (magnification ×20)

The bone substitute Coll/Pro Osteon 200, compared with other biomaterials, gave remarkable aesthetic results, in terms of naturalness and symmetry. These data allowed us to give high VAS scores to patients.^9^ A 3‐year clinical study was performed to evaluate the long‐term results of new bone formation in patients (n = 50) who underwent malar augmentation during orthognathic surgical procedures. The imaging data collected at 1 month after surgery (T1) showed that the prosthesis maintained their granular structure, without evidence of HA granules migration into the surrounding soft tissues (Figure 6A). This result gives evidence of biomaterial physical stability. Stability of implants volume was also observed. In agreement with findings of earlier studies, the size of prosthesis appeared to be stable over time after an initial moderate decrease in volume during the first 18 months.^9^ The prosthesis structure was radiotransparent compared with the compact aspect of the zygomatic bone. At 24 months after surgery (T2), the prosthesis seemed to adhere staunchly to the underlying zygomatic bone in all patients (Figure 6B). The granular structure was still distinguishable, although less evident if compared with the previous healing period. The partial radiotransparency evolved to a radiopacity, similar to that observed in the compact part of the native bone, making it impossible to distinguish the interface between the prosthesis and bone (Figure 6B). At 36 months after surgery (T3), that tendency continued in agreement to the imaging data, toward progressive loss of definition of the granular architecture and an almost complete radiopacity and apparent corticalization of the bone in contact with the prosthesis. The interface between the prosthesis and bone at T3 appeared indistinguishable (Figure 6C).

Histological analysis on bone specimens, harvested from three patients requiring plate device removal 2 years after surgery, was carried out. The persistence of porous HA scaffold and macrophages, although without inflammatory infiltrate, was found in samples. In analyzed fields, fibrous stroma was revealed in 50% of the biopsies, whereas new osteogenesis and mature bone was found in 70% of these specimens (Figure 6D). Immunohistochemical investigations uncovered some cathepsin K protease contained in the cytoplasm of the macrophages, thus indicating the presence of osteoclast activity localized around HA granules (Figure 6D). The anti‐CD56 antibodies indicated a higher amount of new bone formation at the side of the biopsy sample adjacent to the native bone (deep), confirming the results of histomorphometric analyses (data not shown). Specifically, in agreement with the biopsy data, the presence of mature bone was found prominently at the periosteal side, whereas the presence of new immature bone was detected entirely on the deep layer of the native bone with a bone maturation gradient proceeding from the periosteal to the deep side.

## 4 DISCUSSION

The HA‐Coll hybrid scaffold was evaluated using an in vitro cellular model consisting of primary hASCs and a cohort of maxillofacial patients for its osteoinductivity and biocompatibility proprieties and bone regrowth. Epigenetic analyses carried out in 84 genes of the osteogenetic pathway, in hASCs grown on biomaterial, indicate that among DEGs BMP2 is induced and upregulated. Accumulating studies proved that BMP‐2 is involved in bone formation, bone remodeling, bone development, and osteoblast differentiation.^31^, ^32^ BMP‐2, belonging to the BMPs family, is a potent osteoinductive cytokine from the transforming growth factor beta (TGF‐β) family, and it is currently the most commonly used protein‐based bone graft substitute.^33^ Recombinant human bone morphogenetic protein 2 (rhBMP‐2), in human amnion MSCs, with nano‐HA/Coll/poly (l‐lactide), provides a potential approaches in tissue engineering for periodontal bone regeneration.^31^ Furthermore, in vitro and in vivo results using a rat cranial bone defect model demonstrated that the 3D nanofibrous scaffolds incorporated with nano‐hydroxylapatite (nHA) and BMP‐2 peptides exhibited favorable biocompatibility and osteoinductivity.^34^ In addition, the DEG analysis performed by RT^2^ Profiler PCR array allowed us to detect early epigenetic modifications induced in hASCs by scaffold.

In our experiments, the biomaterial modulated ALP gene expression at mRNA level and its enzymatic activity. ALP is a bone‐specific alkaline phosphatase synthesized by the osteoblasts, whereas it is involved in the calcification of bone matrix.

DEGs include the beta‐GLAP gene encoding for the OCN. OCN expression profile and polypeptide correlated with the mRNA and protein levels. Indeed, ELISA data show a statistically significant increase of the OCN in cells grown on biomaterial, at the three time points, that is, at days 14, 21, and 40, compared with the control.

The expression profiles of other osteogenic proteins were not analyzed.

EGFR, which plays important roles in osteogenesis, was upregulated by the biomaterial, up to day 40. EGFR signaling pathway is an important bone regulator, whereas it primarily plays an anabolic role in bone metabolism. The suppression of the IGF‐1R/mTOR‐pathway by the EGFR/ERK/IGFBP‐3 signaling is necessary to balance the osteoblast differentiation/maturation providing a mechanism for the skeletal phenotype observed in EGFR‐deficient mice.^35^ EGFR signaling is important for maintaining osteoprogenitor population at an undifferentiated stage reducing the expression of two major osteoblastic transcription factors, Runx2 and SP7/Osterix, in osteoblast differentiating cells.^36^ Runx2 and SP7 are transcription factors necessary for bone formation and responsible for controlling the differentiation of MSCs in osteoblasts.^37^, ^38^ In our experiments the expression of transcription factors Runx2 and SP7 was upregulated up to day 21, whereas it was not upregulated at day 40. It is possible that the continuous activation of EGFR in undifferentiated osteoprogenitor cells grown on scaffold could attenuate the expression of SP7 and Runx2, found upregulated only at day 21. SPP1 gene that codifies for the osteopontin was modulated by the biomaterial. Osteopontin protein is considered to play an important role in bone regrowth, as well.^39^

Among DEGs, modulated by the biomaterial, there are BMPR1b and SOX9. The knockdown of BMPR1b by siRNA inhibited the osteogenic differentiation of human bone marrow‐derived mesenchymal stem cells^40^; BMPRIB appears primarily expressed in mesenchymal precartilage condensations, in differentiated osteoblasts and chondrocytes.^41^ Sox9 is a transcription factor that plays a key role in chondrogenesis, by controlling the expression of type II collagen and aggrecan, as well as by supporting chondrocyte survival and hypertrophy.^42^ It seems essential for chondrocyte differentiation.

Quantitative reverse transcriptase‐polymerase chain reaction revealed an increased expression of the RANKL, also known as tumor necrosis factor ligand superfamily member 11 (TNFSF11) with an FC (Log2) of 4.36 and FC 4.87 at days 21 and 40, respectively. During the process of the physiological bone remodeling RANKL, the main osteoclastic effector, is expressed in a membrane‐bound form on many mesenchymal cells including MSCs, osteoblasts, osteocytes, and chondrocytes.^43^ To maintain normal bone homeostasis, RANKL signaling must be properly regulated.^43^ Downregulated genes were those that encode for (i) cell‐to‐cell adhesion, such as VCAM1 and COMP, (ii) cell ECM adhesion, such as CD36, ITGAM. These molecules may play a role in the remodeling of the ECM and formation of new histological structures. Interestingly, VCAM1 is continuously downregulated up to day 40.

In hASCs grown on biomaterial CLEC3B protein was found to be expressed up to day 40. CLEC3B protein, which binds Ca^2+^, was investigated because of its potential involvement in the bone mineral metabolism. In hASCs CD105‐enriched cell populations, an increased CLEC3B expression was detected in response to osteoinduction.^30^ In a previous study, we reported that hASCs grown on the biomaterial, in an early phase at days 3 and 9, upregulated specific genes involved in bone mineralization and ossification processes.^17^ It is worth recalling that the osteoinductive activity of the biomaterial is highlighted by the matrix mineralization detected in hASCs grown on the scaffold, up to day 40.

hASC‐eGFP morphology was indistinguishable from parental hASCs. The cytoskeleton architecture seemed to be well organized, whereas its integrity remains uninfluenced by the biomaterial, up to day 40. In addition, SEM analysis carried out on scaffold showed a different histologically bone‐like structure when observed without and with cells grown on it, up to day 40. This difference could depend on the cell matrix produced and released on the scaffold.

The hybrid scaffold used in vivo had a successful outcome in patients, demonstrated by a significant osteogenic induction. The radiologic findings confirmed that the prosthetic material changes over time; initially, it is radiotransparent with a granular structure but over time shows increasing uniformity and radiopacity, which presupposes that the material has been resorbed and replaced by cortical bone. In a follow‐up of 36 months after surgery, the radiologic evaluation showed an almost complete radiopacity and apparent corticalization of the bone in contact with the prosthesis in maxillofacial patients. The expression of protein CD56 indicated a higher amount of new bone formation at the side of the biopsy sample adjacent to the native bone. According to biopsy findings, the presence of mature bone was found preeminently at the periosteal side, whereas the presence of new immature bone was detected entirely on the deep layer of the native bone. CD56 protein in the newly formed bone of maxillofacial patients was in agreement with osteogenic genes found to be upregulated in cellular model of stem cells. The expression of cathepsin K protease contained in the cytoplasm of the macrophages, thus indicating the presence of osteoclast activity localized around HA granules.

Previous studies reported that the pivot role, for hASCs osteogenic differentiation, is played by the HA,^44^, ^45^, ^46^, ^47^ whereas the collagen mainly supports the survival, proliferation, and chondrogenic differentiation.^48^, ^49^

## 5 CONCLUSION

Important biological processes, underlying the continuous supply of hASCs for bone remodeling, were the osteoblastic, chondrogenic, and osteoclastic inductions stimulated by HA‐Coll hybrid scaffold in patients. In conclusion, the hybrid scaffold investigated herein seems to be an excellent biomaterial able to drive bone regrowth and remodeling. This performance is due to Coll/Pro Osteon characteristics which allow to enhance in hASCs the adhesion, morphology, and proliferation, while inducing upregulation of osteogenic genes with improving matrix mineralization and cell viability.

## CONFLICT OF INTEREST

G.W.A. is the assignee of U.S. patent 6506217 B1 (moldable post‐implantation bone filler and method). The other authors indicated no potential conflicts of interest.

## AUTHOR CONTRIBUTIONS

A.D'A., L.T.: involved in experimental planning and interpreting data, performed the surgery, wrote the manuscript; E.M., F.M., M.T.: involved in experimental planning and interpreting data, wrote the manuscript; M.G., G.W.A.: performed the surgery; I.B., M.R.I., J.C.R.: performed the array experiments; S.P., C.G.: developed the in vitro model of hASC fluorescent engineering with recombinant adenovirus.

## Supporting information


**Table S1** List of genes found to be up‐regulated and down‐regulated in hASCs grown on the scaffold at day 21

**Table S2** List of genes up‐regulated and down‐regulated in hASCs grown on the scaffold at day 40.

## Data Availability

The data that support the findings of this study are available from the corresponding author upon reasonable request.
