# Supplementary material for: Hydroxylapatite‐collagen hybrid scaffold induces human adipose‐derived mesenchymal stem cells to osteogenic differentiation in vitro and bone regrowth in patients
Source: Stem Cells Transl Med. 2019 Dec 13;9(3):377–88. doi: 10.1002/sctm.19-0170 (PMC7031637; doi:10.1002/sctm.19-0170)
Supplement: Supplementary file 1 — Table S1 List of genes found to be up‐regulated and down‐regulated in hASCs grown on the scaffold at day 21 [file SCT3-9-377-s001.docx]

**Table S1. List of genes found to be up-regulated and down-regulated in hASCs grown on the scaffold at day 21**

Up-regulated genes Down-regulated genes

Number Symbol/ Fold-Change Number Symbol/ Fold-Change

Acronym (Log_2_ FC) Acronym (Log_2_ FC)

1 ALP 1.15 1 VCAM1 -1.66

2 BGLAP 1.94

3 BMP1 1.20

4 BMP2 1.62

5 BMPR1B 2.78

6 COL1A1 1.05

7 CSF2 3.41

8 CSF3 5.18

9 EGFR 1.37

10 FGFR1 1.04

11 FGFR2 1.35

12 IGF1 1.90

13 IGF1R 1.06

14 NOG 1.75

15 RUNX2 1.59

16 SOX9 1.18

17 SP7 4.89

18 SPP1 1.88

19 TGFB1 1.51

20 TNFSF11 4.36

21 TWIST1 1.27

22 VDR 1.33

Alkaline Phosphatase (ALP), Bone Gamma-Carboxyglutamate (gla) (BGLAP), Bone Morphogenetic Proteins 1 and 2 (BMP1 and 2); Bone Morphogenetic Protein Receptor type IB (BMPR1B); Collagen type I alpha 1 (COL1A1); Colony Stimulating Factor 2 and 3 (CSF2 and 3); Epidermal Growth Factor receptor (EGFR); Fibroblast Growth Factor Receptor 1 and 2 (FGFR1 and 2); Insulin Growth Factor 1 (IGF1) and its Receptor (IGF1R); Noggin (NOG); Runt-related Transcription Factor 2 (RUNX2); Transcription Factor SOX9 (SOX9), Transcription Factor Sp7 (SP7), Secreted Phosphoprotein 1 (SPP1), Transforming Growth Factor Beta 1 (TGFB1); TNF Superfamily Member 11 (TNFSF11); Twist Family BHLH Transcription Factor 1 (TWIST1); Vitamin D Receptor (VDR); Vascular Cell Adhesion Molecule 1 (VCAM1).
