# Supplementary material for: Hydroxylapatite‐collagen hybrid scaffold induces human adipose‐derived mesenchymal stem cells to osteogenic differentiation in vitro and bone regrowth in patients
Source: Stem Cells Transl Med. 2019 Dec 13;9(3):377–88. doi: 10.1002/sctm.19-0170 (PMC7031637; doi:10.1002/sctm.19-0170)
Supplement: Supplementary file 2 — Table S2 List of genes up‐regulated and down‐regulated in hASCs grown on the scaffold at day 40. [file SCT3-9-377-s002.docx]

**Table S2**. **List of genes up-regulated and down-regulated in hASCs grown on the scaffold at day 40.**

Up-regulated genes Down-regulated genes

Number Symbol/ Fold-Change Number Symbol/ Fold-Change

Acronym (Log_2_ FC) Acronym (Log_2_ FC)

1 BMP2 1.89 1 BMP6 -1.14

2 BMPR1B 3.73 2 CD36 -1.33

3 CSF2 2.73 3 COMP -2.16

4 CSF3 4.88 4 FGFR2 -2.11

5 EGF 1.47 5 IGF2 -2.15

6 EGFR 1.04 6 ITGAM -7.12

7 ITGA2 2.20 7 NOG -1.05

8 SOX9 1.75 8 TGFB3 -1.07

9 SPP1 2.84 9 VCAM1 -1.83

10 TNFSF11 4.87

Bone Morphogenetic Protein 2 (BMP2); Bone Morphogenetic Protein Receptor type IB (BMPR1B); Colony Stimulating Factor 2 and 3 (CSF2 and 3); Epidermal Growth Factor (EGF); Epidermal Growth Factor Receptor (EGFR); Integrin Subunit Alpha 2 (ITGA2); Transcription Factor SOX9 (SOX9), Secreted Phosphoprotein 1 (SPP1), TNF Superfamily Member 11 (TNFSF11); Bone Morphogenetic Protein 6 (BMP6); CD36 Molecule (CD36); Cartilage Oligomeric Matrix Protein (COMP); Fibroblast Growth Factor Receptor 2 (FGFR2); Insuline-like Growth Factor 2 (IGF2); Integrin Subunit Alpha M (ITGAM); Noggin (NOG); Transforming Growth Factor, Beta 3 (TGFB3); Vascular Cell Adhesion Molecule 1 (VCAM1).
